# Supplementary material for: Physical activity and sedentarism among seniors in France, and their impact on health
Source: PLoS One. 2022 Aug 18;17(8):e0272785. doi: 10.1371/journal.pone.0272785 (PMC9387786; doi:10.1371/journal.pone.0272785)
Supplement: Attachment — Submitted filename: Response to Reviewers.docx [file pone.0272785.s001.docx]

Response to Reviewers

We would like to begin by thanking the experts warmly for their valuable criticism. We have taken all their remarks into account in our revised version, and here in this supporting letter would like to explain what changes we have made to facilitate its reading.

| **Comments from Reviewers** | **Responses form the authors** |
| --- | --- |
| Please modify the title to ensure that it is meeting PLOS’ guidelines (<https://journals.plos.org/plosone/s/submission-guidelines#loc-title>). In particular, the title should be "specific, descriptive, concise, and comprehensible to readers outside the field" and in this case we feel it is not informative and specific about your study's scope and methodology. | We have modified the title in accordance with the guidelines’ recommendations so that it is informative with regard to the aims and methodology (Physical activity and sedentarism among seniors in France, and their impact on health). |
| We note you have included a table to which you do not refer in the text of your manuscript. Please ensure that you refer to Table 6 in your text; if accepted, production will need this reference to link the reader to the Table. | The reference to Table 6 has been included in the text. |
| Is the manuscript presented in an intelligible fashion and written in standard English? | The manuscript has been corrected by an English-speaking expert. |
| **Reviewer #1** | |
| - in Table 5 and elsewhere, please use 'housework' or 'domestic work' rather than housekeeping - unsure of use of 'point up' (denotes emphasis. 'point to' would suffice here - Getting the public (no plural), or if you want to emphasise multiplicity, then use different population groups - Avoid the use of 'the elderly' - Seniors or older adults is preferable - use full stops for decimal points (not commas). | The text has been altered in response to these remarks. |
| Requiring clarification: - unsure what you mean by 'parent population' - total population? | We have specified that reference is made to the general population aged 55-74 (we have used 'total population' rather than 'parent population'). |
| - Reference to questionnaire administered face-to-face and self-administered. This isn't clear. Was the questionnaire delivered to people by someone who waited while respondents filled in it themselves. Or was the questionnaire in two parts, one part administered by a researcher and the other self-completed? | We have clarified that it was a self-administered questionnaire completed in the presence of a researcher. |
| - what do you mean by 'complex sampling design'? | An explanatory reference has been added. |
| - I cannot find a special issue of Ageing in Society 2012. There is no journal with this title. However, I think that Ageing & Society published a digital special issue on later life and physical activity. Is this what you mean? | This has been corrected to Ageing & Society. |
| *Comments about the text:* | |
| There is no reference to increasing recognition that the WHO PA recommendations are being discussed in the PA literature with a need identified to expand them with requirements to engage in strength/resistance, as well as balance and aerobic work. This reference might be useful 10.1136/bjsports-2018-100451 | We have added the suggested reference, which perfectly illustrates the discussions on PA prescriptions. We thank the reviewer for this suggestion. |
| I think the construction of a PA & SB typology should be signposted as an objective of the analysis. This typology enables you to integrate PA and SB into a more fine-grained understanding of older adults' practices and risks. This makes your analysis distinctive and should be promoted more forcefully in the discussion as well. | We have added this objective to the abstract and the introduction and have also included it in the conclusion to give it more strength, as suggested by the reviewer. |
| Your discussion should return to gender differences - your findings appear to reflect the gendering of behaviours and dispositions as normative which culminate in greater or lesser propensity to PA & SB as men and women age. This is important. | A sub-section to highlight gender differences has been added in the Typology section. |
| You claim in your conclusion that a third of older adults are sedentary - did you combine Type 1 and Type 2 (ie not PA & SB + not PA and not SB) to arrive at this claim? I wasn't sure. Please make explicit. | We have clarified this point in the conclusion, but also in certain passages of the text, so that the profiles we are referring to are perfectly clear. We have numbered the profiles in Table 9 for easier and clearer reference. The title of Table 9 was not clear: we have added "SB" in the title so no doubt remains. |
| You almost dismiss the finding that just over half are active and non-sedentary. That's quite an encouraging finding and perhaps should be highlighted as much as the disappointing % of non active, at risk older adults. | We have highlighted this heartening finding more in the abstract but also in the conclusion, which begins with this aspect of the results. We have changed the title, which was too discouraging and did not reveal the content of the article (old title: “A third of French seniors physically inactive”). |
| **Reviewer #2** | |
| The study provides population-based estimates of self-reported physical activity and sedentary behaviour among older adults in France, and explores some of the potential sociodemographic and health correlates. The data are fairly recent (2014-2016), but things have likely become very different in recent years due to the Covid pandemic. This has been overlooked in the Discussion. | The conclusion now makes reference to the consequences of the pandemic, cites a reference on its impact on seniors and suggests further study to measure the consequences of the pandemic on the physical activity of older adults. |
| English language editing is recommended – especially consistency of tense. | We have had the text reread by an English-speaking specialist. |
| There are some referencing errors: eg in the paragraph “Screens: an activity popular among adults” there is a superscript reference as well as within-square-bracket references. | Checks have been carried out and modifications made. |
| *Abstract:* | |
| Study design unclear – longitudinal, cross-sectional? | The fact that the study is cross-sectional has been made clear. |
| Aim – very broad, without any actual aim/research question/hypothesis | The aims have been clarified. |
| Results – what does the P value refer to – women vs men? | For greater clarity, the P value has been removed from the abstract, and it is now explained in the text. |
| Results are not very informative | Greater clarity has been given to the most striking results: general results, the differences between men and women, and the 4 profiles. |
| Significant links with health indicators? Unclear which ones, in which way. | The different indicators and most significant ones have been clarified. |
| Conclusion – cause and effect cannot be determined by these data and analyses | The conclusion has been reformulated. |
| *Main paper:* | |
| Intro – PA definition is any movement, but then look at guidelines which are exclusively based on MPA/VPA | We deal with the different forms of PA (sports and leisure physical activities, methods of active transport, domestic physical activities). |
| Need reference for “these recommendations remain valid throughout life and are even more important with advancing age, which is generally associated with increasing vulnerability” | A reference has been added in relation to this point. |
| How were MET values for the physical activities from the survey determined? | The reference to the MET calculation method has been added  (Ainsworth et al., 2011). |
| Presentation of study population descriptive statistics should be the first section of | As suggested, we have altered the plan to make this the first part of the results. |
| Comparison with other studies/populations and discussion of potential mechanisms should occur in Discussion, or rename this section “Results and Discussion” | The plan has been altered to a "Results and Discussion" section as suggested and the sections and sub-sections have been numbered for greater clarity. |
| Change in PA over age – Authors say this is likely due to the deterioration in health, but there are no results presented to show deterioration in health with age. | A reference has been added to substantiate this point. |
| Fig 1 Legend is in French language. | This has been corrected. |
| Throughout paper text and Tables: when presenting p values, unless p<0.001, please present exact values rather than “ns” or <0.01. | In our statistical analyses we prefer to keep only the 3 reference thresholds (*p<0.05; **p<0.01; ***p<0.001) rather than putting the exact value, especially for "ns". This is of course possible but it makes the tables very dense. |
| The wording “highest level of low PA” is a bit confusing. Suggest “lowest levels of PA” | This has been corrected as per the reviewer’s suggestion. |
| Overall comment on Results: a lot of repetition of what is already presented in the Tables. | We have tried to be less repetitive. |
| Conclusion – again, can’t say that you have measured effects on health. From the Results, there appears to only be one health indicator associated with sedentarism, which is obesity. I suggest the authors sharpen their aims so that they can present a clearer conclusion (and more concisely present Results) to address their aims/research questions. | We have focused the Conclusion on the essential elements, linking them more closely with the aims. We have been more specific with regard to the health indicators. |

We wish to thank the experts once again for their constructive criticism and hope that this revised version will meet with your approval.

The authors remain at your disposal for any questions you may have.

Yours sincerely
